# Highly Sensitive Square Wave Adsorptive Stripping Voltammetric Determination of Dopamine in Human Plasma Using a Cytosine-Modified Pencil Graphite Electrode

**DOI:** 10.1021/acsomega.5c02061

**Published:** 2025-05-28

**Authors:** Şeyma Korkmaz, Ayşen Demir Mülazımoğlu

**Affiliations:** † Chemistry Department, Institute of Science, 226846 Necmettin Erbakan University, 42090 Konya, Turkey; ‡ Chemistry Department, Ahmet Keleşoğlu Education Faculty, Necmettin Erbakan University, 42090 Konya, Turkey

## Abstract

Dopamine (DA) is an essential neurotransmitter in the central nervous system, playing a vital role in the human brain. A simple, facile, fast, and low-cost method based on the cytosine-modified pencil graphite electrode (CT/PGE) was developed to determine dopamine (DA) by using the square wave adsorptive stripping voltammetry (SWAdSV) technique. The cytosine (CT) was modified on the bare pencil graphite electrode (PGE) by conducting cyclic voltammetry (CV) over a potential range between +0.7 and +1.9 V for 10 cycles. A number of techniques including CV, electrochemical impedance spectroscopy (EIS), and field-emission scanning electron microscopy (FE-SEM) were used to characterize the CT/PGE. The impact of varying pH values (6.4, 6.8, 7.2, 7.6, and 8.0) and phosphate buffer solution (PBS) on the DA determination was studied using the square wave voltammetry (SWV) technique. The basic voltammetric techniques SWV, SWAdSV, differential pulse voltammetry (DPV), and differential pulse adsorptive stripping voltammetry (DPAdSV) were used to determine the most suitable method for analytical applications. The accumulation time was optimized using the SWAdSV technique on the CT/PGE, within a potential range of −0.4 to +0.4 V. The CT/PGE was evaluated in the presence of several interferents, such as urea, ascorbic acid, and uric acid. Under optimum conditions, the CT/PGE exhibited a well-defined linear relationship for DA across the concentration ranges of 0.1 mM to 0.5 μM and 0.1 μM to 7.5 nM, with a limit of detection (LOD) of 2.28 nM and a limit of quantification (LOQ) of 6.85 nM. The suggested method was effectively applied to the determination of DA in human plasma serum samples to evaluate its suitability.

## Graphical abstract

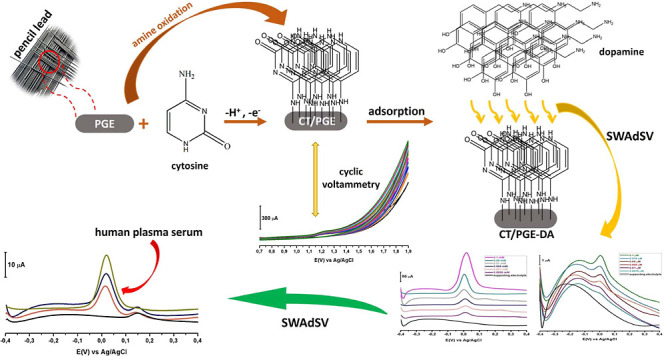

## 1 Introduction

Dopamine (DA), also known as 3,4-dihydroxyphenyl ethylamine, is a neurotransmitter of significant physiological and medical importance.^1^ Belonging to the catecholamine and phenethylamine families, DA is a bioactive molecule that plays a crucial role in the human body, functioning as a neurotransmitter involved in cognitive processes and the regulation of diseases within the central and peripheral nervous systems.^2^ The skeletal formula of DA is depicted in Figure 1. DA is a compound involved in movement control within the basal ganglia, particularly in regulating behavioral and physiological processes, such as movement, reward, learning, motivation, and pleasure. The determination of DA levels is critical in the diagnosis and monitoring of neurological diseases, especially Huntington’s^3^ and Parkinson’s disease.^4^ Increased DA levels can enhance reward- and motivation-based learning mechanisms.^5^

**Figure 1:**
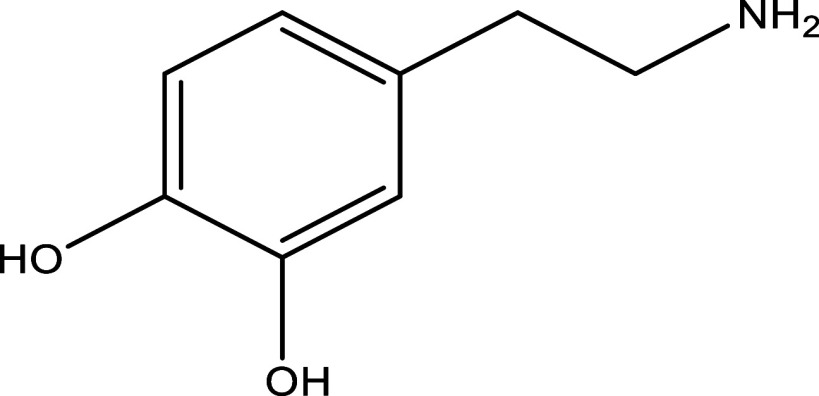
Molecular structure of DA.

DA is also available as an intravenous drug that acts on the sympathetic nervous system, producing effects such as increased heart rate and blood pressure. A deficiency in DA levels in the human brain can lead to conditions such as schizophrenia,^6^ depression,^7^ and diseases like Alzheimer’s^8^ and Parkinson’s.^4^ On the other hand, elevated DA levels may contribute to hypertension, drug addiction, and heart failure.^9^

The concentration of DA in certain human fluids, such as human plasma serum and urine, is very low. Moreover, the complex nature of biological samples containing DA further complicates its measurement, requiring analysts to perform sample pretreatment to achieve accurate quantitative results. In real biological samples, some substances with chemical properties similar to those of DA can cause interference. However, by leveraging DA’s electrochemical, biological, or optical properties, it is possible to selectively determine its concentration, even in small quantities.^10^ Recent advances in research hold great potential for previously held great and improved treatment processes with new technologies and methods that enable more sensitive DA determination.

Modification of carbon surfaces through electrochemical techniques plays a significant role in materials science and electrochemistry.^11–14^ Different modified electrodes are used in electrochemical methods to measure organic and inorganic substances in a wide range of analytical situations.^15–17^ Electrochemical techniques have become increasingly preferred in recent years due to their advantages such as speed, low cost and simplicity equipment, chemical inertness, low background current, wide potential window, and minimal sample utilization.

The electrochemical oxidation of amine derivatives represents a widely utilized and effective strategy for the fabrication of chemically modified electrodes (CMEs). In this process, amines are oxidized at the electrode surface to form reactive radical cations, which subsequently participate in the formation of covalently bonded films or single layer films. Notably, the modification of aromatic amines yields conductive and functional surface coatings that substantially enhance the sensitivity and selectivity of electrochemical sensors. The surface modification method not only is simple, cost-efficient, and environmentally benign but also improves electrode performance by enabling specific interactions with biomolecules or metal ions, thereby broadening the scope of electroanalytical applications.^18–21^

Voltammetric studies often utilize carbon-based electrodes, including glassy carbon electrodes,^22^ pencil graphite electrodes (PGE),^23^ carbon paste electrodes,^24^ and graphene.^25^ PGEs exhibit properties such as disposability, high electrical conductivity, chemical stability, and inertness, as well as the ability to operate over a wide potential range. They are cost-effective, easily available, practical, and simple to use. The disposable nature of PGEs helps prevent contamination in experiments, ensuring precise measurements. Their ability to operate over a wide electrochemical potential range allows reduction and oxidation reactions to be carried out at different potential intervals.^26–30^

DA was determined using conventional methods such as chromatography,^31^ colorimetry,^32^ chemiluminescence,^33^ fluorescence,^34^ and spectrophotometry.^35^ These conventional methods present disadvantages such as expensive equipment requirements, complex sample preprocessing, and time consumption. Electrochemical methods like cyclic voltammetry (CV),^36,37^ linear sweep voltammetry (LSV),^38,39^ square wave voltammetry (SWV),^40,41^ differential pulse voltammetry (DPV),^42,43^ electrochemical impedance spectroscopy (EIS),^44^ square wave adsorptive stripping voltammetry (SWAdSV),^45,46^ and second-order derivative linear sweep voltammetry^47,48^ were used to find DA.

This study aims to develop a highly sensitive and selective voltammetric sensor for the determination of DA in human plasma serum using a cytosine-modified pencil graphite electrode (CT/PGE) in combination with SWAdSV. The main objectives are to

(i) Fabricate a disposable and cost-effective electrochemical platform by modifying the PGE with CT,
(ii) Characterize the CT/PGE using CV, EIS, and field-emission scanning electron microscopy (FE-SEM),
(iii) Determine the optimum pH for DA detection,
(iv) Identify the most suitable voltammetric technique to achieve a low detection limit and wide linear dynamic range,
(v) Evaluate the sensor’s performance by testing its selectivity against common interferences such as ascorbic acid, urea, and uric acid,
(vi) Validate the applicability of the proposed method by measuring DA in human plasma serum with high recovery.

## 2 Materials and Methods

### 2.1 Instruments and Apparatus

A potentiostat/galvanostat/ZRA (Gamry, USA, model reference 600+) was used for the application of cyclic voltammetric, linear sweep voltammetric, square wave voltammetric, square wave adsorptive stripping voltammetric, differential pulse voltammetric, and differential pulse adsorptive stripping voltammetric excitation to measure the resulting current and potential regimes. The same device was used for EIS in the characterization study. All of the experiments were executed using a three-electrode electrochemical cell system. The reference electrodes utilized were Ag/AgCl/3 M KCl (BASi, USA, model MF-2056) in an aqueous medium and Ag/Ag^+^/10 mM AgNO_3_ (BASi, USA, model MF-2062) in a nonaqueous medium. A platinum wire (BASi, USA, model MW-1032) was employed as an auxiliary electrode. 0.7 mm diameter Faber Castel (2B) brand PGEs were employed as the working electrode in bare and modified forms. FE-SEM images were utilized to characterize the bare and the modified electrode using ZEISS, Germany, model Gemini 500. All water-based solutions were made with ultrapure water that has a resistivity of 18.2 MΩ cm (MP MINIPURE Purification System, DEST UP, USA) and used as received.

### 2.2 Chemicals and Reagents

All chemicals in this research were of analytical-grade purity and used directly without any further treatment. DA, cytosine (CT), and additional chemicals were supplied by Sigma-Aldrich, Riedel, and Merck. Human plasma serum was purchased from Sigma-Aldrich. By mixing K_2_HPO_4_ and KH_2_PO_4_ standard stock solutions, phosphate buffer solution (PBS) with different pH levels was made. A VWR pHenomenal pH 1100L pH meter (VWR, UK) with a combined glass pH electrode was used to measure the pH levels in water-based solutions. 0.1 M NaOH was used to adjust the pH.

### 2.3 The Analyzed Samples Preparation

Human plasma serum samples acquired from Sigma-Aldrich were kept in a freezer at −70 °C until analysis. 50 μL of human plasma serum samples were diluted to 20 mL with pH 7.2 PBS^49^ and 5 mL of standard DA solutions were added. pH 7.2 PBS (pH 7.2) and 0.1 M NaOH were employed to set the pH to 7.2, achieving a final volume of 25 mL for the sample solution.

## 3 Results and Discussion

### 3.1 Electrochemical Modification of CT on the Bare PGE Surface

Electrochemical modification is a crucial step in electroanalytical studies, as it allows for the development of sensor electrodes specific to the species to be determined, which can then be used selectively. This study investigated the detectability of DA by modifying the bare PGE surface with CT and using various electroanalytical techniques. To achieve this, a 1 mM CT solution was prepared in PBS pH 7.2, and modification of the bare PGE surface was performed using CV. The modification was carried out within a potential range of +0.7 to +1.9 V, with a scanning rate of 100 mV s^–1^ for 10 cycles. Before characterization and analytical determinations, CT/PGE was washed with appropriate supporting electrolytes and prepared for analysis.

The successful application of the modification process is evident from the voltammogram shown in Figure 2, where the oxidation of the –NH_2_ functional group present in the CT structure was demonstrated. The primary aim of the multicycle modification process is to prevent small gaps, known as pinholes, from remaining on the electrode surface. The secondary aim is to achieve a more uniform stacking of the modifier species on the electrode surface. To this end, the modification process was performed over multiple cycles. Based on the evaluation of the obtained data, the optimum number of cycles was determined to be 10.

**Figure 2:**
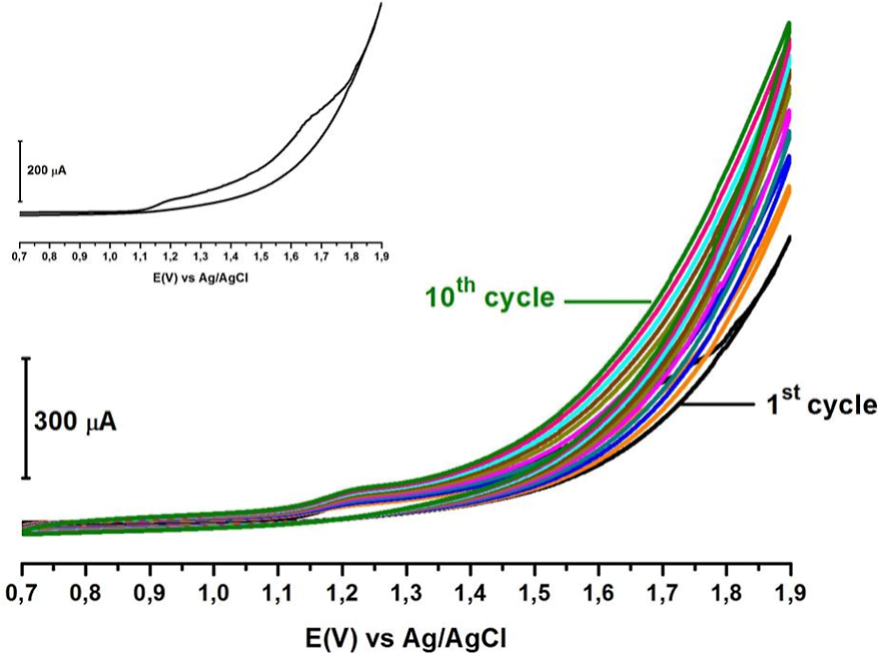
Cyclic voltammogram of modification of 1 mM CT in PBS pH 7.2 versus Ag/AgCl/3 M KCl on the bare PGE surface at the potential range +0.7 to +1.9 V using a 100 mV s^–1^ scanning rate for 10 cycles.

During the electrochemical modification of an electrode surface via amine oxidation, an initial anodic scan is performed to oxidize the species in the atomic, ionic, or molecular form present in the solution. In molecules containing amine functional groups, this process typically involves the loss of one proton (H^+^) and one electron (e^–^), resulting in the formation of a radical at an amine group. This radical species becomes highly reactive and ready for covalent attachment to the electrode surface. The subsequent step involves covalent binding of the radical species to the electrode, which occurs within the electrical double layer near the electrode interface. As a result, the original electrode surface becomes coated with the modifying molecule, yielding a newly functionalized surface.^13,41^ As illustrated in Figure 3, the proposed mechanism begins with the oxidation of the modifying agent, CT, specifically at its amine groups. Subsequently, the CT molecule, transported to the electrode surface via a diffusion-controlled process, forms a covalent C–N–C bond with the surface, thereby completing the grafting process.

**Figure 3:**
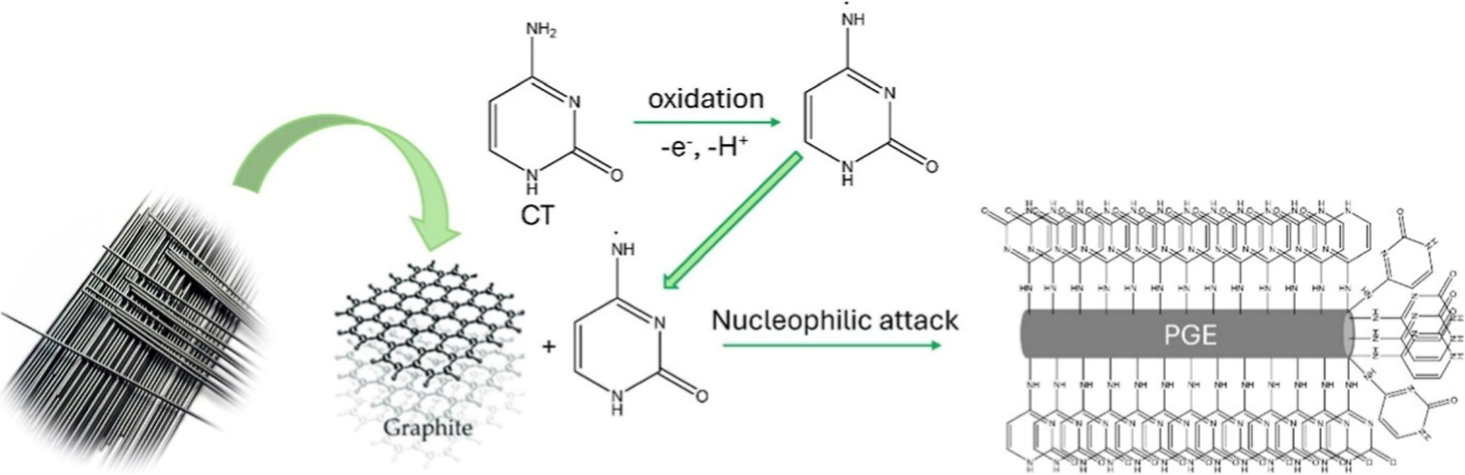
Mechanism of CT grafting onto the PGE surface.

### 3.2 Surface Characterization Studies with CV, EIS, and FE-SEM

Various electrochemical, microscopic, and spectroscopic techniques are available for carrying out characterization processes in electroanalytical studies. After the modification process, it is essential to investigate the usability of the new electrode in different media. In voltammetric studies, characterization processes are typically performed in both aqueous and nonaqueous media. In this way, voltammograms obtained with different redox probes in different media are compared to assess whether the modified electrode is suitable for the study. In this study, voltammetric characterization was performed in both nonaqueous and aqueous media using ferrocene (FCN) and hexacyanoferrate III (HCF­(III)) redox probes. Characterization studies conducted with the CV technique involved the use of a 1 mM FCN redox probe solution prepared in 100 mM tetrabutylammonium tetrafluoroborate (NBu_4_BF_4_) dissolved in acetonitrile (CH_3_CN), with an Ag/Ag^+^ reference electrode, in a nonaqueous medium. The procedure involved a positive scan followed by a negative scan within the potential range of 0.0 V to +0.5 V. The cyclic voltammograms of the bare PGE and the CT/PGE were obtained and overlaid for comparison (Figure 4A). A redox probe solution of 1 mM HCF­(III) was made in 100 mM H_2_SO_4_ with an Ag/AgCl reference electrode in an aqueous medium. The procedure consisted of a negative scan and then a positive scan within the potential range of +0.4 to 0.0 V. The cyclic voltammograms of the bare PGE and the CT/PGE were superimposed and compared (Figure 4B).

**Figure 4:**
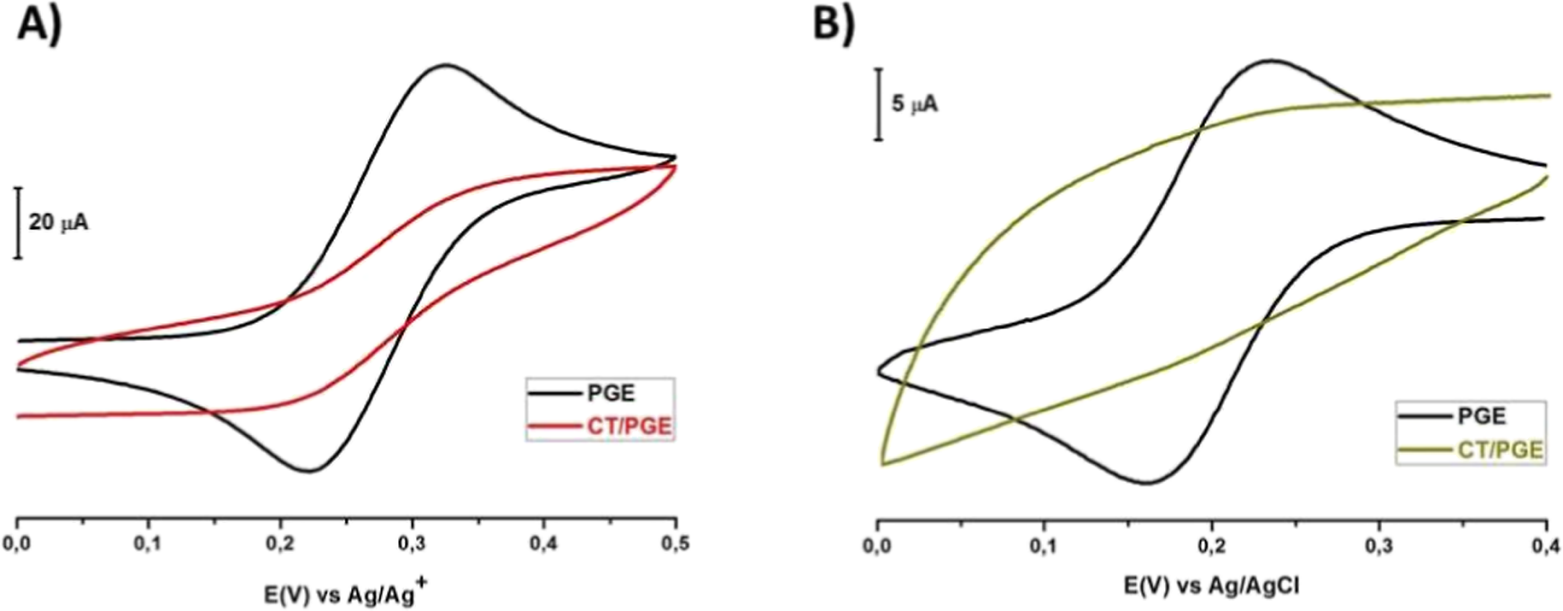
Superimposed image of cyclic voltammograms: (A) 1 mM FCN redox probe solution in 100 mM NBu_4_BF_4_ (in CH_3_CN) versus Ag/Ag^+^ (10 mM AgNO_3_) at a 100 mV s^–1^ scanning rate and (B) 1 mM HCF­(III) redox probe solution in 100 mM H_2_SO_4_ versus Ag/AgCl/3 M KCl using a 100 mV s^–1^ scanning rate taken on the bare PGE and the CT/PGE surfaces for one cycle.

In Figure 4A, during positive scanning, Fe^2+^ ions in the FCN solution were oxidized to Fe^3+^ ions, and then Fe^3+^ ions were reduced back to Fe^2+^ ions on the bare PGE surface. In contrast, no peak was observed for the oxidation of Fe^2+^ ions to Fe^3+^ ions and the reduction of Fe^3+^ ions to Fe^2+^ ions in the FCN solution on the CT/PGE surface after 10 cycles. This indicated the bare PGE surface was modified with CT and the modified surface was electroinactive.

Figure 4B, while Fe^3+^ ions in HCF­(III) solution were reduced to Fe^2+^ ions and then Fe^2+^ ions were oxidized to Fe^3+^ ions on the bare PGE surface by negative scanning. After 10 cycles of CT modification, no reduction peak of Fe^3+^ ions in HCF­(III) solution to Fe^2+^ ions was observed, and then oxidation of Fe^2+^ ions to Fe^3+^ ions was observed on the CT/PGE surface. Similar to FCN voltammograms, the bare PGE surface was modified with CT, and it was concluded that the modified surface was electroinactive.

Subsequent to the electrochemical characterization with the CV technique, impedance measurements were performed by EIS using a 1 mM Fe­(CN)_6_^3–/4–^ redox probe mixture prepared in a 100 mM KCl supporting electrolyte. EIS parameters were applied as follows: Initial frequency of 100.000 Hz, final frequency of 0.01 Hz, constant potential applied during the process of 10 mV ms^–1^, and initial delay of 100 s. Figure 5 shows a comparison of the Nyquist plots between the CT/PGE and the bare PGE. Upon examination of the Nyquist plots in Figure 5, minimal resistance was revealed on the bare PGE surface. In contrast, the CT/PGE surface showed significantly higher resistance. Nyquist plots supporting the voltammetric surface characterizations showed that the CT-modified PGE surface was electroinactive.

**Figure 5:**
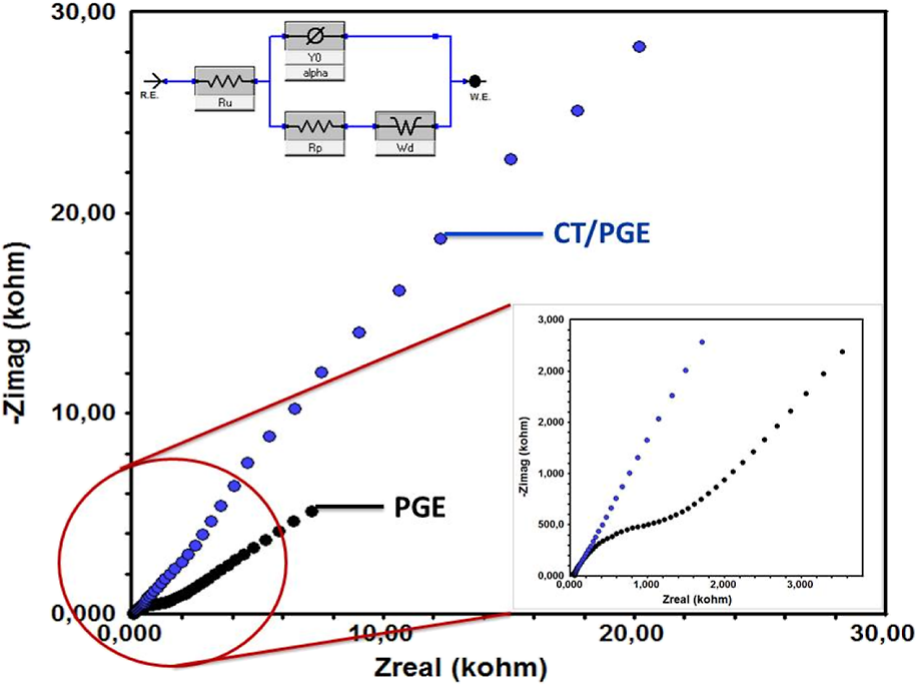
Nyquist plots for EIS of the bare PGE and the CT/PGE surface in a 1 mM Fe­(CN)_6_^3–^/^4–^ redox couple solution in 100 mM KCl at a frequency range 100.000–0.01 Hz and 10 mV wave amplitude.

As a result of the simulation, considering the equivalent circuit proposed for the Nyquist plots in Figure 5, the *R*_ct_ value for the bare PGE surface was determined to be 1.18 × 10^3^ ± 2.94 Ω. After the modification process, the *R*_ct_ value increased compared to the value calculated for the CT/PGE, reaching 960 × 10^3^ ± 0.028 Ω. The higher *R*_ct_ value of the modified surface compared to the bare surface indicates that the CT/PGE creates an electroinactive surface for electron and mass transfer, preventing the diffusion of Fe^2+^/Fe^3+^ ions toward the electrode surface. The parameters calculated for the CT/PGE surface after impedance measurements were as follows: *R*_ct_ = 960 × 10^3^ ± 0.028 Ω, *R*_s_ = 52.0 ± 1.32 Ω, *Y*_o_ = 69.0 × 10^–6^ S, and α = 0.75.

The surface area (*A*) of the PGE, possessing a cylindrical structure, was calculated using eq 1. The *A* value calculated here was then substituted in eq 2 to calculate the *k* ^0^ values.

1 $$A=2\pi rh+2\pi r^{2}$$

Equation 2 was used to calculate the electron transfer rate constant, *k* ^0^, for both the bare PGE and the CT/PGE surfaces.

2 $$R_{ct}=\frac{RT}{{\left(nF\right)}^{2}Ak^{0}C}$$

In eq 1, *A* represents the electrode area (cm^2^); *n* is the number of electrons required for the oxidation/reduction of Fe^2+^/Fe^3+^ ions in the Fe­(CN)_6_^3–^/Fe­(CN)_6_^4–^ mixed solution; *C* (mol cm^–3^) is the molar concentration of the Fe­(CN)_6_^3–^/Fe­(CN)_6_^4–^ solution; *R* (8.314 J mol^–1^ K^–1^) is the ideal gas constant; *T* (K) is the temperature; and *F* (96.485 C mol^–1^) is the Faraday constant.^50^ Using this equation, the calculated *k* ^0^ values for the bare PGE and the CT/PGE surfaces were 1.918 × 10^–3^ cm s^–1^ and 2.357 × 10^–6^ cm s^–1^, respectively.

3 $$\theta =1-\left(\frac{k_{CT/PGE}^{0}}{k_{PGE}^{0}}\right)$$

By substitution of the *k* ^0^ values calculated in eq 2 into eq 3, the coating efficiency of the modified surface, θ, was determined to be 99.87%.

Even when the modified electrode surface is not electroactive, determination can be achieved through adsorption onto the surface. The crucial factor is the selective interaction between the modifying agent and the analyte, allowing the modified electrode to function as a sensor electrode. Similar to many electrochemical determination studies in the literature, DA detection in this study was performed via adsorption on the electrode surface.^14,51^

In addition to the electrochemical characterization studies, microscopic images of the modified surfaces were obtained using the FE-SEM technique at magnifications of 5.00 k×, 10.00 k×, and 20.00 k×. The FE-SEM images of the bare PGE and the CT/PGE at a 5.00 k× magnification, obtained by scanning approximately 4 μm areas, in Figure 6A,B, revealed the morphological changes resulting from the modification of the bare PGE with CT. As observed in the 10.00 k× magnification in Figure 6C and the 20.00 k× magnification in Figure 6D, the CT layers are typically stacked in thin layers.

**Figure 6:**
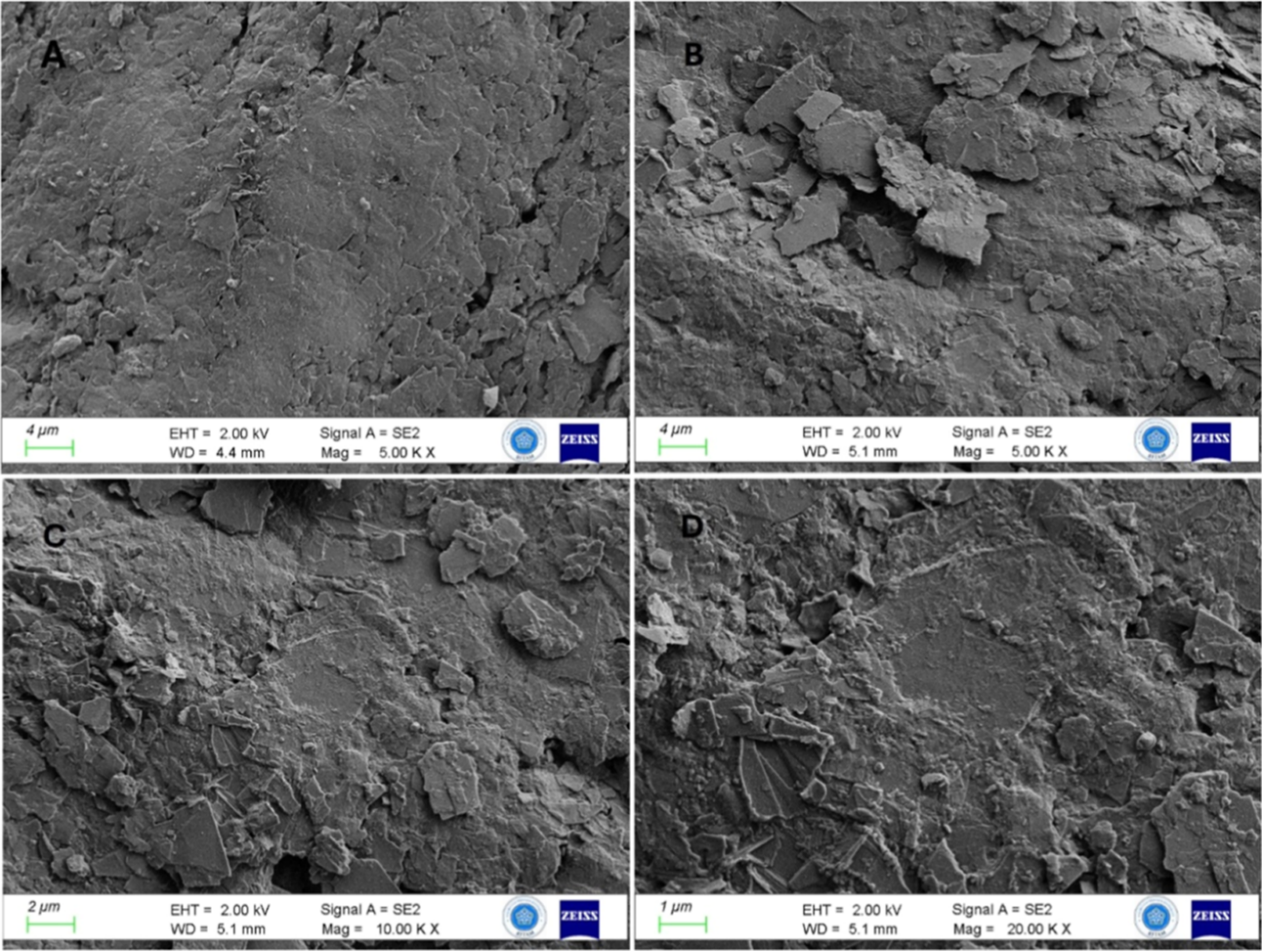
FE-SEM images for (A) the bare PGE surface magnification of 5.00 k×, (B) the CT/PGE surface magnification of 5.00 k×, (C) the CT/PGE surface magnification of 10.00 k×, and (D) the CT/PGE surface magnification of 20.00 k×.

### 3.3 Influence of Scan Rate

The superimposed voltammograms obtained by the LSV technique in Figure 7A were used to demonstrate whether the binding of the CT molecule to the bare PGE surface in solution occurs by potential application and was diffusion-controlled. The Randles–Sevcik equation (eq 4) is used in voltammetric studies and demonstrates the linear relationship of peak currents with both the square root of the scan rate and the concentration.

4 $$I_{p}=268.600n^{3/2}AD^{1/2}C\nu^{1/2}$$

**Figure 7:**
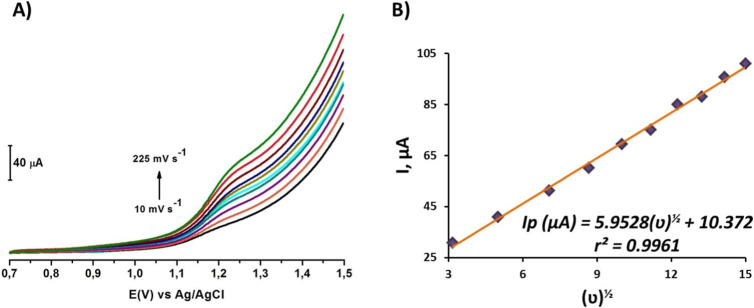
(A) LS voltammograms of 1 mM CT in PBS pH 7.2 versus Ag/AgCl/3 M KCl on the bare PGE surface at the potential range +0.7 to +1.5 V at varying scan rates of 10, 25, 50, 75, 100, 125, 150, 175, 200, and 225 mV s^–1^. (B) The graph of peak current against square root of scanning rate.

To this end, voltammograms were obtained using the LSV technique at varying scanning rates (10, 25, 50, 75, 100, 125, 150, 175, 200, and 225 mV s^–1^) with 1 mM CT containing PBS at pH 7.2, within the potential range from 0.7 to 1.5 V, and were superimposed and compared in Figure 7A. Based on the Randles–Sevcik equation, as shown in Figure 7B, a linear relationship with respect to the peak currents and the square roots of the scan rates was observed, suggesting the diffusion-controlled transport of CT to the bare PGE surface.

### 3.4 Effect of the Supporting Electrolyte pH

An optimization study was conducted using 1 mM DA solutions prepared in PBS at varying pH values (6.4, 6.8, 7.2, 7.6, and 8.0), and SW voltammograms were obtained in the potential range from −0.4 to +0.4 V using the SWV technique. Upon examining the SW voltammograms shown in Figure 8A, it was found the oxidation peak current for 1 mM DA was maximal at pH 7.2, and a negative shift in the oxidation peak potential of DA was observed as the pH increased, which aligns with the literature. Based on this behavior, it seems that at pH 7.4, proton participation in the electrode reaction was predominant for DA.^52^ DA has a p*K*_a_ value of 8.72, according to some reports. For fully ionizing a weakly basic compound, the pH should be two units below the p*K*_a_ value.^53^ Therefore, based on the peak current values obtained from Figure 8B, pH 7.2 PBS was selected for further studies.

**Figure 8:**
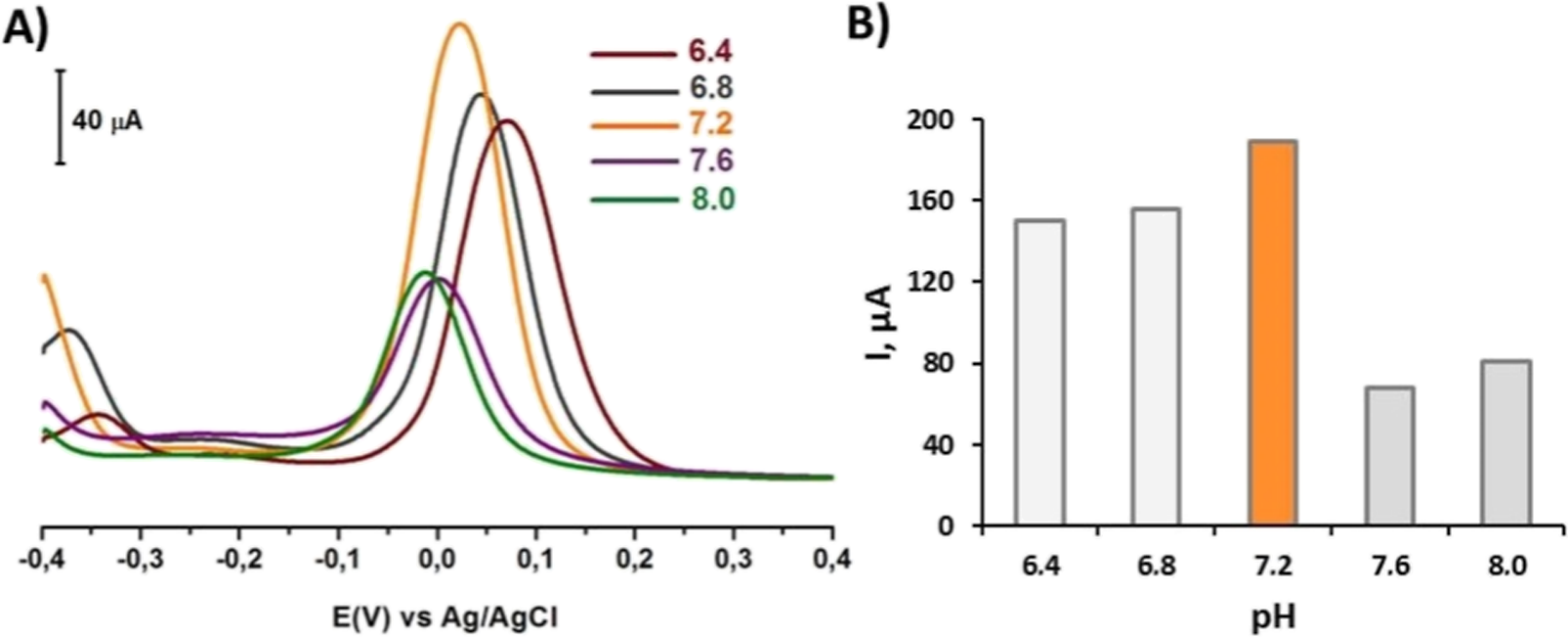
(A) SW voltammograms recorded on the CT/PGE at the potential range +0.7 to +1.9 V using a 1 mM DA solution prepared at various pH levels in 0.1 M PBS. (B) Corresponding plot of the peak current versus pH.

### 3.5 Selection of the Optimum Voltammetric Method

The SWV, DPV, SWAdSV, and DPAdSV measurements were carried out in 1 mM DA at pH 7.2 PBS. All experiments used the CT/PGE as the working electrode and the Ag/AgCl electrode as the reference electrode^12^ for the optimum voltammetric method. The SW voltammogram was made using the SWV method, which has a pulse size of 50 mV, a frequency of 25 Hz, and a step size of 1 mV in the potential range of −0.4 V to +0.4 V (Figure 9A). The SWAdS voltammogram was obtained with the SWAdSV technique using a pulse size of 50 mV, frequency of 25 Hz, step size of 1 mV, and accumulation time of 60 s within the potential range from −0.4 V to +0.4 V (Figure 9B). The DP voltammogram was obtained by the DPV technique using 25 mV pulse size, 1 mV step size, and 0.1 s pulse time in the potential range from −0.4 V to +0.4 V (Figure 9C). The DPAdS voltammograms were acquired with the DPAdSV technique within the potential range from −0.4 V to +0.4 V using 25 mV pulse size, 1 mV step size, 0.1 s pulse time, and 60 s accumulation time (Figure 9D).

**Figure 9:**
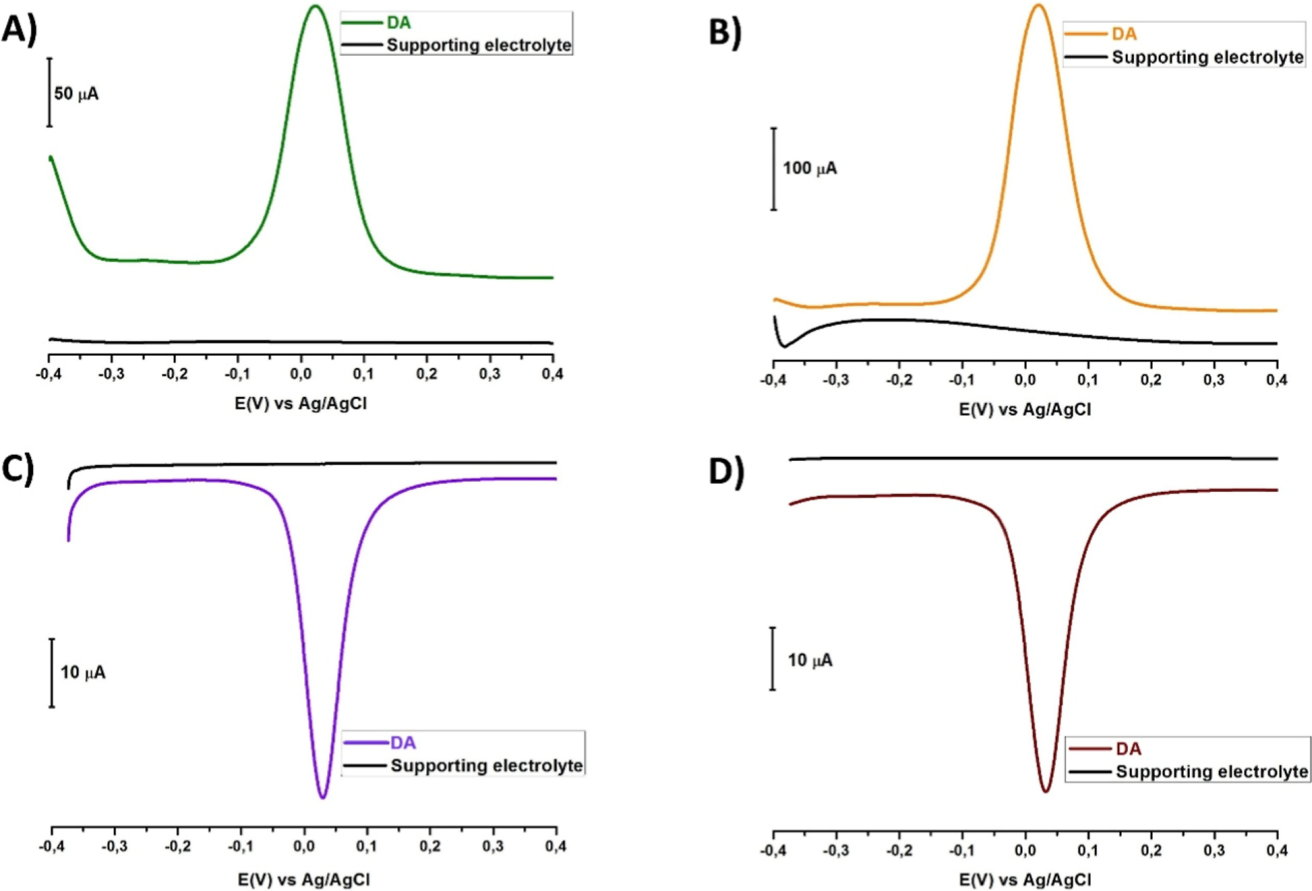
(A) SWVs, (B) SWAdSVs, (C) DPVs, and (D) DPAdSVs taken in the potential range from −0.4 V to +0.4 V using a 1 mM DA solution prepared in pH 7.2 PBS containing 1 mM DA versus Ag/AgCl/3 M KCl on using the CT/PGE.

In comparison to the voltammograms in Figure 9A–D, the peak current was 188.07 μA with the SWV technique, 317.30 μA with the SWAdSV technique, 46.58 μA with the DPV technique, and 47.42 μA with the DPAdSV technique. Subsequent studies achieved DA detection using the SWAdSV technique and a pH 7.2 PBS supporting electrolyte solution.

### 3.6 The Effect of Accumulation Time

The first of the two main steps in the SWAdSV technique is the deposition of DA on the CT/PGE surface, and the second is the stripping of the surface material. The SWAdS voltammogram was obtained with the SWAdSV technique using a pulse size of 50 mV, a frequency of 25 Hz, and a step size of 1 mV within the potential range from −0.4 V to +0.4 V. The SWAdSV technique was used to measure the accumulation time at different times (0, 15, 30, 45, 60, 75, 90, 105, 120, 135, and 150 s) and potentials (−0.4 V to +0.4 V). This was done to determine the best accumulation time. The SWAdS voltammograms of 1 mM DA in pH 7.2 PBS on the CT/PGE were compared, as shown in Figure 10A, and the peak current versus the accumulation time was plotted in Figure 10B. The accumulation times applied for DA were analyzed, and the highest peak intensity was found to be at 90 s.

**Figure 10:**
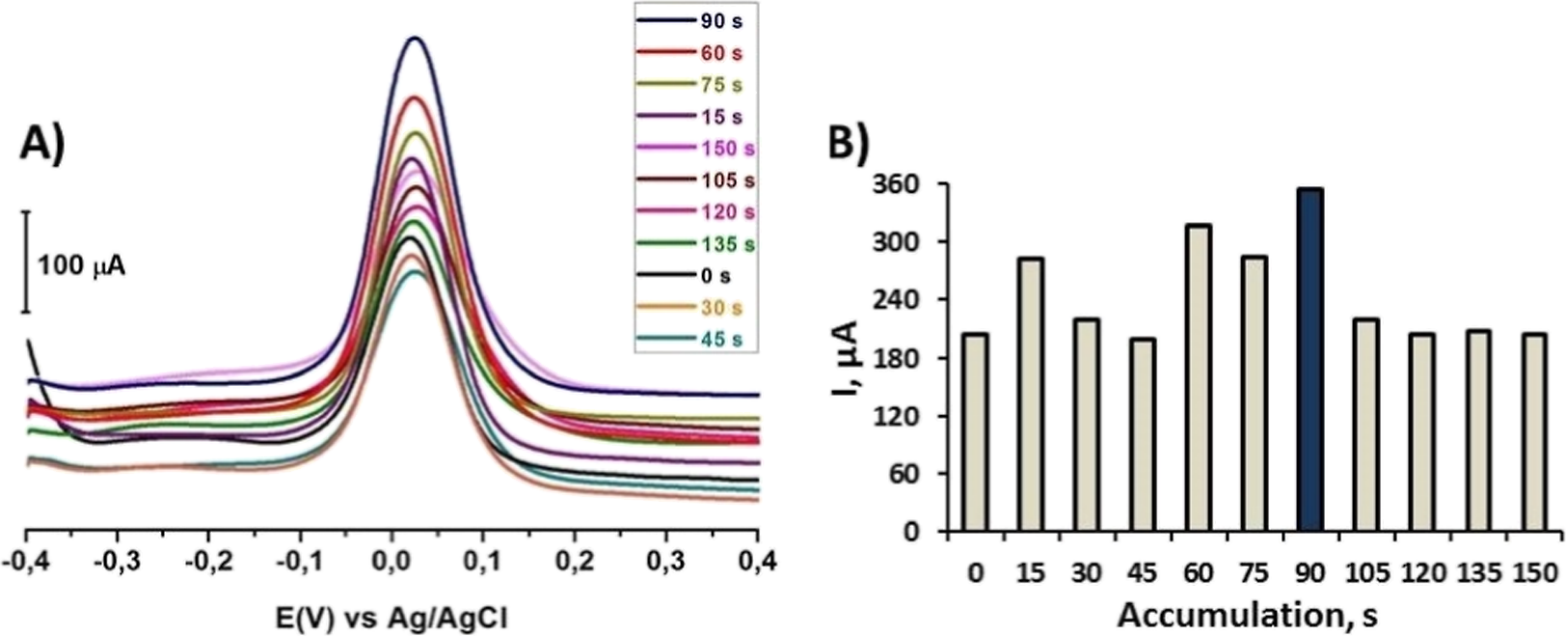
(A) SWAdS voltammograms of 1 mM DA in PBS at pH 7.2 for various accumulation times (0, 15, 30, 45, 60, 75, 90, 105, 120, 135, and 150 s) recorded at the CT/PGE at the potential range from −0.4 V to +0.4 V, frequency 25 Hz, pulse size 50 mV s^–1^, step size 1 mV, and stirring rate 400 rpm. (B) Corresponding plot of the peak current (μA) versus the accumulation time (s).

### 3.7 Effect of Interferences

Determining method selectivity is of great importance in chemical analysis. This study investigated how interfering compounds affect DA measurements. 100 μM ascorbic acid, 100 μM urea, and 100 μM uric acid were added separately as interfering compounds to a 10 μM DA solution prepared in pH 7.2 PBS and voltammograms were taken using the SWAdSV technique within the potential range of −0.4 V to +0.4 V. Ascorbic acid, which plays a crucial role as an antioxidant in the body, is an inherent interfering compound for DA within the biological nervous systems and in uric acid.^54^

Figure 11 showed the effect of the presence of uric acid, ascorbic acid, and urea on the voltammetric analysis of DA by the SWAdSV technique. The results showed no significant effect on the peak currents of DA even at a 10-fold higher concentration of interferents.

**Figure 11:**
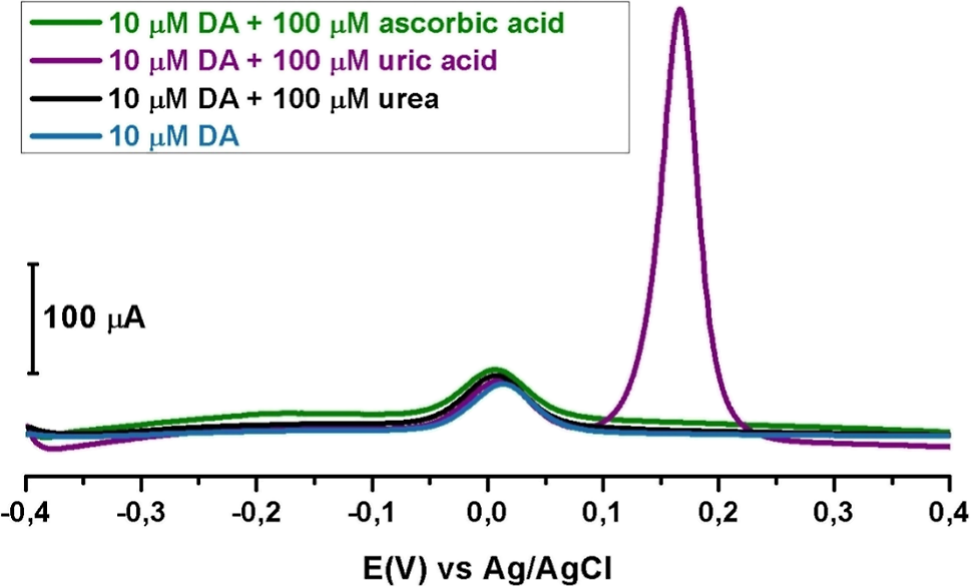
SWAdS voltammograms for the CT/PGE in pH 7.2 PBS (containing (blue) 10 μM DA, (black) 10 μM DA + 100 μM urea, (purple) 10 μM DA + 100 μM uric acid, and (green) 10 μM DA + 100 μM ascorbic acid) at the potential range from −0.4 V to +0.4 V. Frequency 25 Hz, pulse size 50 mV s^–1^, step size 1 mV, stirring rate 400 rpm, and accumulation times 90 s.

### 3.8 Reproducibility Studies of the CT/PGE for DA

In this study, repeatability measurements were carried out using a 0.1 mM DA solution. The evaluation of the data was based on the adsorption of DA onto the CT-modified PGE surface by using the SWAdSV technique. As a result of the analysis, the relative standard deviation (% RSD) was calculated to be 0.5142 for five repeated measurements. This low % RSD value indicates a high level of repeatability for the measurements. Based on the results of the stability and repeatability studies, it was concluded that the CT/PGE electrode is a suitable sensor for the determination of DA in the sample matrix. Figure 12 presents the overlaid voltammograms obtained from the five repeated measurements along with the proposed reaction mechanism for the adsorption of DA molecules onto the CT/PGE surface.

**Figure 12:**
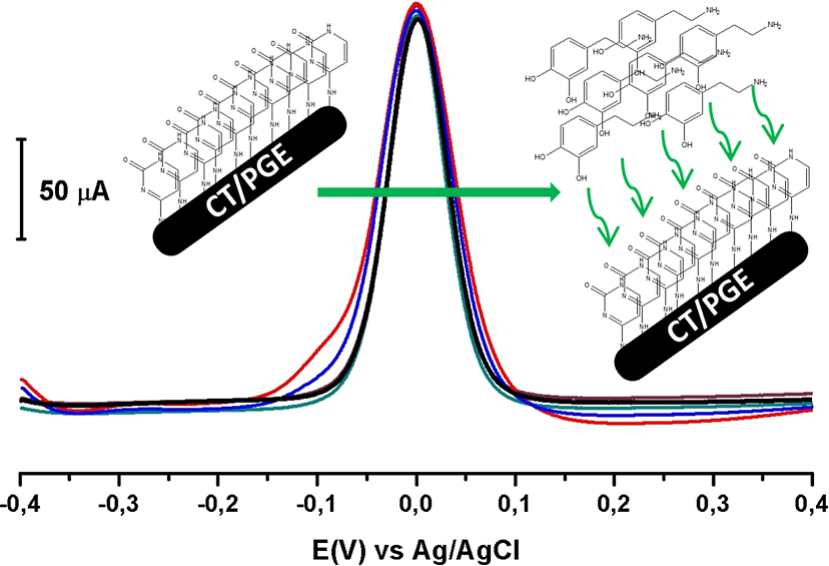
Overlaid voltammograms obtained from the repeatability study performed in five replicates and the proposed adsorption reaction mechanism. SWAdS voltammograms of the CT/PGE in pH 7.2 PBS 0.1 mM DA at the potential range from −0.4 V to +0.4 V.

### 3.9 Electrochemical Performance toward DA

The CT/PGE was tested toward DA using the SWAdSV technique with an accumulation time of 90 s at pH 7.2 PBS. Using peak currents obtained from Figure 13A, the calibration graph in Figure 13B was plotted with DA solutions prepared between 0.1 mM and 0.5 μM. Based on the peak currents from Figure 13C, the calibration graph in Figure 13D was plotted with lower concentrations between 0.1 μM and 7.5 nM. Subsequently, the slope of the linear equation corresponds to the sensor’s sensitivity value. The calibration curve showed that the anodic peak current (*I*_pa_) and the concentration increased linearly. This led to two linear equations: *I*_p_ (μA) = 1772.1*C*_DA_ (mM) + 21.074 in Figure 13B and *I*_p_ (μA) = 9.5124*C*_DA_ (μM) + 0.0433 in Figure 13D. The correlation coefficients for these equations were 0.9967 and 0.9973, respectively.

**Figure 13:**
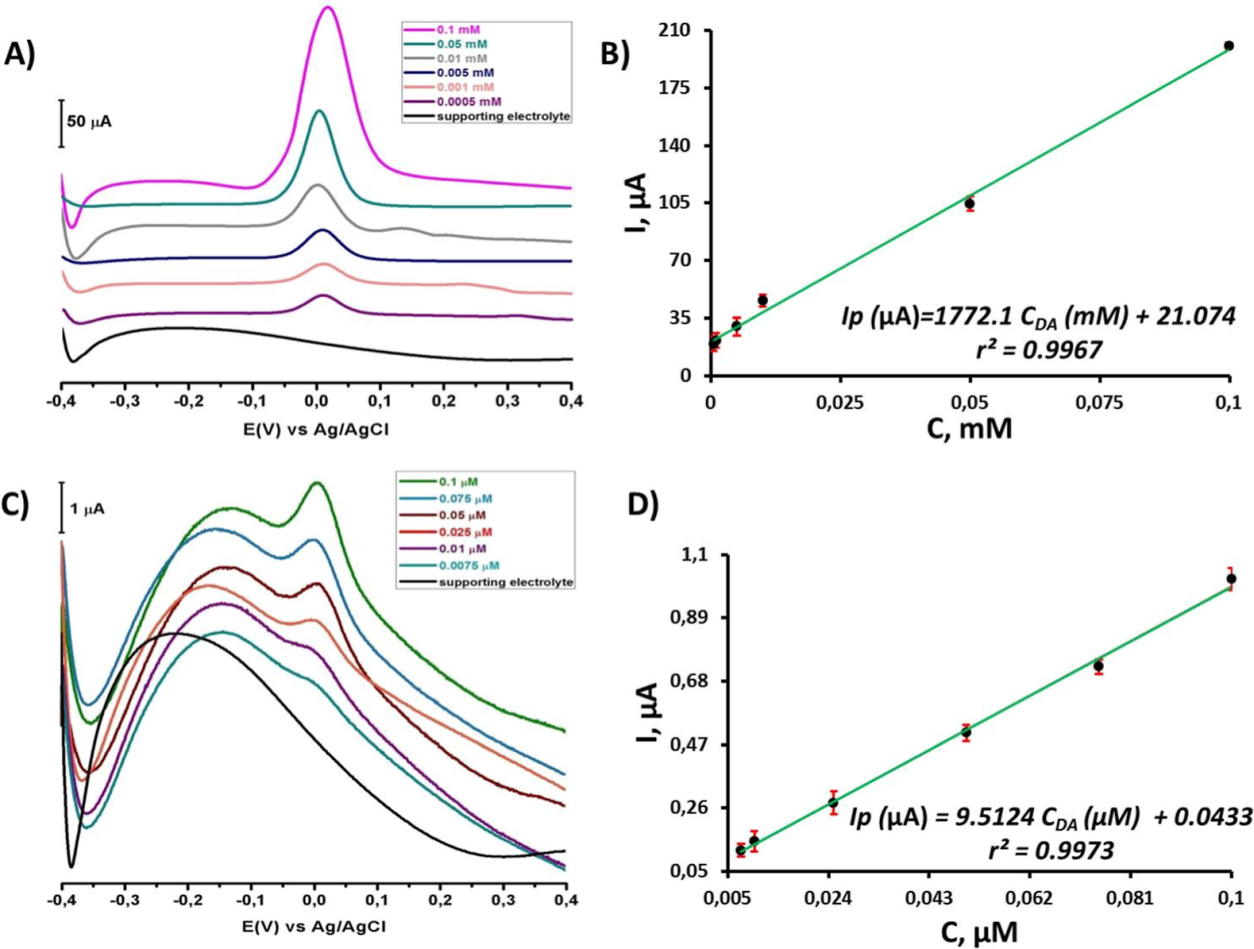
(A) SWAdS voltammograms of DA at various concentrations (0.1, 0.05, 0.01, 0.005, 0.001, and 0.0005 mM) at the CT/PGE surface at the range from −0.4 V to +0.4 V. Frequency 25 Hz, pulse size 50 mV s^–1^, step size 1 mV, stirring rate 400 rpm, and accumulation times 90 s. (B) The linear plot for peak current and concentration of DA. (C) SWAdS voltammograms of DA at various concentrations (0.1, 0.075, 0.05, 0.025, 0.001, and 0.0075 μM) at the CT/PGE surface at the range from −0.4 V to +0.4 V. Frequency 25 Hz, pulse size 50 mV s^–1^, step size 1 mV, stirring rate 400 rpm, and accumulation times 90 s. (D) The linear plot for peak current and concentration of DA.

The limit of detection (LOD), calculated by using eq 5, is a crucial performance metric for a sensor, representing the lowest detectable concentration of DA relative to background noise.

5 $$LOD=3.3\frac{\sigma }{S}$$

6 $$LOQ=10\frac{\sigma }{S}$$

The standard deviation of the background noise (σ) is determined by taking five SWAdSV measurements of the background signal in the supporting electrolyte in pH 7.2 PBS without DA. Additionally, *S* is the slope of the linear calibration curve. The factor 3.3 in eq 5 gives the standard signal-to-noise ratio. The limit of quantification (LOQ) in eq 6 is similarly calculated with a multiplication factor of 10.

The LOD and LOQ were determined to be 2.28 and 6.85 nM, respectively. These low values demonstrate the high sensitivity of the method for DA detection.

According to Table 1, Fan and co-workers reported that a SGPGE was effective for the detection of DA in real human urine samples up to 1.5 μM due to the fabrication of the PGE into a high-performance sensing material.^55^ Kaya and colleagues utilized novel pS-BIL MIP/PeGE, which was subjected to different DA concentrations. The determination of the analyte, relying on its oxidation peak, was carried out using the sensitive electroanalytical method of DPV, showing a low detection limit of 20 nM and a broad linear range of DA concentrations from 0.05 μM to 250 μM.^56^ Devaramani et al. detected DA at low concentrations using the electrografting of 4-ABSA onto the GPLE.^57^ Shashikumara and co-workers revealed that DA in injection samples was detected with enhanced recovery and sensitivity using PGEs modified with poly­(yellow PX4R). The method’s high sensitivity, coupled with its improved selectivity, represents a significant advancement for the molecular diagnosis of Parkinson’s disease.^58^ Sankaranarayanan and Venkateswaran reported using an anodized PGE to detect DA and uric acid at low concentrations. The good recovery rates for DA in human blood serum and uric acid in urine samples showed that an APGE could be used in point-of-care analysis.^59^ Bahrami et al. used a new voltammetric biosensor made by adding Cu nanoparticles to PGEs and found that the LOD for DA determination was 1.07 μM.^60^

**Table 1:** Comparison of the Present Study with Previous Related Research on the Determination of DA Using Voltammetry^a^

voltammetric technique	electrode	LOD (μM)	linear range (μM)	pH/buffer solution	reference
DPV	SGPGE	0.0082	0.15–45	7.0 (PBS)	^55^
DPV	pS-BIL MIP/PeGE	0.02	0.05–250	7.4 (PBS)	^56^
DPV	4-ABSA/GPLE	0.095	0.5–10.0	7.0 (PBS)	^57^
CV	poly(yellowPX4R)/MPGE	3.05	10–60	7.4 (PBS)	^58^
DPV	APGE	0.008	1–80	7.0 (PBS)	^59^
DPV	Cu/Cu_*x*_O NPs/PGE	1.07	0.3–53	5.8 (PBS)	^60^
SWAdSV	CT/PGE	0.00228	0.0075–100	7.2 (PBS)	**this work**

a Surface-graphenized pencil graphite electrode (SGPGE), polymer (pS-BIL MIP)/pencil graphite electrode (PeGE), 4-aminobenzene sulfonic acid/graphite pencil lead electrode (4-ABSA/GPLE), poly­(yellow PX4R)/pencil graphite electrode (poly­(yellow PX4R)/MPGE), anodized pencil graphite electrode (APGE), and pencil graphite electrodes modified by Cu/Cu_*x*_O nanoparticles (Cu/Cu_*x*_O NPs/PGE).

### 3.10 Utilization of the CT/PGE in Human Serum Sample

To assess the accuracy of the proposed method and the validity of the obtained calibration graph, DA was sequentially added to the prepared human serum samples at concentrations of 1.0, 5.0, and 10 μM. Using the SWAdSV technique under optimized conditions, voltammograms were recorded within a potential range of −0.4 V to +0.4 V, and the overlaid voltammograms are presented in Figure 14. Based on the obtained results, recovery (%) and relative standard deviation (%) values are provided in Table 2. According to Table 2, the DA concentrations were determined as 0.99, 5.02, and 10.13 μM, with recovery rates of 99.82%, 100.14%, and 101.29%, respectively. These findings confirm the applicability and reliability of the method for the DA determination.

**Figure 14:**
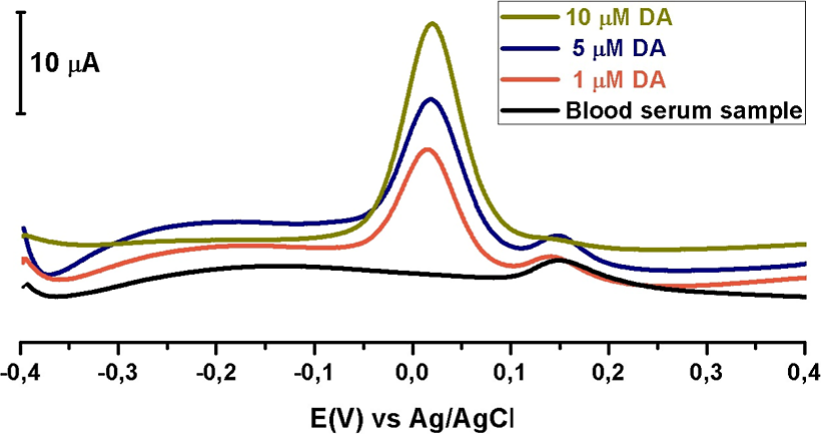
SWAdS voltammograms of human plasma serum sample containing 10 μM, 5 μM, and 1 μM concentration DA in PBS pH 7.20 at the CT/PGE surface at the potential range of −0.4/+0.4 V. Frequency 25 Hz, pulse size 50 mV s^–1^, step size 1 mV, stirring rate 400 rpm, and accumulation times 90 s.

**Table 2:** Percentage Recovery Results for Serum Samples with DA Standard Solutions by Applying the CT/PGE (*N* = 3)

added (μM)	found (μM)	recovery (%)	relative standard deviation (%)
10.0	10.13	101.29	±0.049
5.0	5.02	100.14	±0.078
1.0	0.99	99.82	±0.076

## 4 Conclusions

The disposable and low-cost bare PGE was modified with CT, and for the first time, the CT/PGE was used for DA determination in all studies. In the current study, CV, EIS, and FE-SEM techniques were used to describe the sensor electrode that was made by changing the CT molecule on the bare PGE surface.

The disposability and easy availability of the PGE made the work faster and cheaper. The influence of pH on the peak current was examined for DA determination using the SWV technique with the CT/PGE sensor electrode in a supporting electrolyte solution. After the optimum pH of 7.2 was determined, the feasibility of DA determination was explored using different voltammetric techniques. Subsequent studies using the SWAdSV technique demonstrated the ability to determine DA at very low concentrations.

Urea, ascorbic acid, and uric acid did not affect the DA determination. LOD and LOQ were determined using the calibration curve plotted for DA determination without any pretreatment. Previous studies using the SWAdSV technique for DA determination were limited. The suggested methodology was effectively utilized to determine DA in human plasma serum samples, offering a cost-effective, rapid, and selective approach.
